# Agonist-induced Piezo1 activation promote mitochondrial-dependent apoptosis in vascular smooth muscle cells

**DOI:** 10.1186/s12872-022-02726-2

**Published:** 2022-06-24

**Authors:** Qing Yin, Guangyao Zang, Nannan Li, Chenchen Sun, Rongzeng Du

**Affiliations:** 1 Department of Cardiology, Affiliated Hospital of Jiangsu University, Zhenjiang, 212001 Jiangsu Province China; 2 School of Medicine, Jiangsu University, Zhenjiang, 212001 Jiangsu Province China

**Keywords:** Piezo1, Apoptosis, Ca^2+^ overload, Mitochondrial destruction

## Abstract

**Objective:**

Mechanical damage plays an essential role in the progression of atherosclerosis. Piezo1 is a new mechanically sensitive ion channel. The present study investigated the vascular smooth muscle cells (VSMCs) apoptosis induced by Piezo1 activation and explored its underlying mechanism.

**Methods:**

We evaluated cell viability and apoptosis rate with cell counting kit-8 (CCK-8) and Annexin V-FITC/PI flow cytometry assay, respectively. And then Western blot was performed to measure the relative protein. Reactive oxygen species (ROS) and intracellular Ca^2+^ were assessed via fluorescence microscope, and the mitochondrial transmembrane potential was monitored by JC-10 staining.

**Results:**

Our in vitro study revealed that mice in the ApoE-/- group compared with control mice showed higher Piezo1 expression(*P* < 0.05). Besides, Yoda1, a Piezo1 agonist, triggered Ca^2+^ overload, mitochondrial damage, accumulation of ROS, and VSMCs apoptosis in a dose-depend manner. Furthermore, BAPT-AM (an intracellular Ca^2+^ chelator) and NAC (an antioxidant) suppressed the mitochondrial damage and attenuated the VSMCs apoptosis.

**Conclusion:**

Our study suggested that Piezo1 induced VSMCs apoptosis because of Ca^2+^ overload, excessive ROS generation, and mitochondrial dysfunction, which indicated that Piezo1 has potential value in treating vascular diseases.

**Supplementary Information:**

The online version contains supplementary material available at 10.1186/s12872-022-02726-2.

## Introduction

Vascular smooth muscle cells are the main components of blood vessels and the main body of atherosclerotic plaque formation. The imbalance of apoptosis and proliferation of VSMCs causes various vascular pathological states, especially the apoptosis of vascular smooth muscle, leading to plaque instability, aneurysm formation, and post-stent restenosis[1–3]. Previous studies have found that in atherosclerosis, the construction of vulnerable plaque is mainly caused by the decrease of VSMCs and the increase of macrophages[4]. Simultaneously, compared with stable plaque, the apoptosis level of VSMCs in vulnerable plaque is upregulated. Therefore, VSMCs apoptosis plays a vital role in the development of atherosclerosis. The factors that cause vascular smooth muscle cell apoptosis include the secretion of pro-inflammatory cytokines, oxidative stress, and mechanical damage.[5]. The mechanism of vascular smooth muscle cell apoptosis induced by mechanical injury is not yet precise.

As a mechanosensitive ion channel, Piezo1 senses the mechanical stimulation of blood vessels by local blood flow and converts them into chemical signals to regulate various biological processes such as inflammation and angiogenesis during atherosclerosis. Piezo1 senses changes in local blood vessels and transmits these changes to cells and causes changes in cell function. It has been found that Piezo1, which exists on endothelial cells, can be activated at the turning point of the aortic inner wall, where the blood flow shear force changes the most, and activate integrin α6 and FAK in a Piezo-Gq/G11-dependent manner, It further promotes the activation of NF-κB pathway and promotes the occurrence of atherosclerosis. In addition, it can also be regulated at the gene level, with Piezo1 activation increasing the expression of pro-atherosclerotic genes[6]. Therefore, when applying force on the cells, Piezo1 can quickly cause calcium to enter the cells. Piezo1 expressed in platelets was found to cause the import of Ca^2+^ and thrombus formation under arterial shear force[7]. Besides, Piezo1 has been found in type II lung cells. The mechanical stretching of the alveolar induced by acute respiratory distress syndrome (ARDS) activates the Piezo1 channel, resulting in calcium injection causing type II lung cell apoptosis[8]. Piezo1 controls the progression of intervertebral disc degeneration by inducing nucleus pulposus cell inflammation through the Ca^2+^/NF-κB pathway [9]. All studies have shown that Piezo1 facilitates the ions to enter the cell, transforming mechanical signals into chemical signals.

Therefore, our study aimed to investigate the role of Piezo1 in VSMCs on atherosclerotic plaque formation with its special agonist Yoda1 and to study the potential mechanism of Piezo1 mediating vascular smooth muscle cell apoptosis. Our work might provide a new target for the prevention and treatment of vascular diseases.

## Materials and methods

### Animals

All animal experiments have been approved by the Experimental Animal Use Ethics Committee of Jiangsu University and comply with Directive 2010/ 63/EU guidelines. HFD group: ApoE^−/−^ mice (6–8 week of age; Vital River laboratories, Distributor of Jackson Laboratory, Beijing, China) were fed with a high-fat diet (41 kcal% fat and 43 kcal% carbohydrates), and NC group: C57BL/6 J mice (Experimental Animal Center of Jiangsu University) were provided with a normal diet and water ad libitum. The mice were anesthetized utilizing isoflurane inhalation, and subsequently, the aortas were carefully dissected for the following experiments.

### Hematoxylin–eosin (HE) and immunofluorescence staining

The aortas were harvested from the C57 or ApoE^−/−^mice. Next, a 4% paraformaldehyde solution was used to fix tissues for 24 h. The samples were paraffin-embedded and cross-sectioned (5 μm) for hematoxylin and eosin (H&E) staining. The VSMCs were stained with the primary antibody of α-SMA and secondary antibody of goat anti-mouse IgG Alexa Fluor® 488 and anti-Piezo1 antibody, and secondary antibody of goat anti-rabbit IgG Alexa Fluor® 647. Cell nuclei were stained with 4',6-diamidino-2-phenylindole (DAPI), and five slides were created for each group and repeated at least six times. Detailed information on antibodies and chemicals was shown in Additional file 1: Supplementary Table S1.

### Cell culture

The VSMCs were obtained from the thoracic aorta of male, eight-week-old C57BL/6 J mice, cultured in high glucose Dulbecco's modified Eagle's medium (DMEM) supplemented with 20% fetal bovine serum (Gibco, USA), 100 U/mL penicillin, and 100 mg/mL streptomycin (Gibco, USA) and cultured at in humidified 5% CO_2_ plus 95% O_2_ atmosphere at 37℃. The culture medium was changed 48 h after the start of plating, and experiments were initiated when the cells in the plate wall showed 80%. α-SMA Fluorescence Staining of Smooth Muscle Cells was shown in Additional file 1: Supplementary Figure S1.

### CCK-8 assay

First, 100μL of cells suspension containing 5 × 10^3^ cells were put into each well of the 96-well plate and incubated at 37℃ for 12 h in a 5% CO_2_ humidified atmosphere. The cells were incubated with serum-free DMEM for 12 h for synchronization before the experiment. The culture medium was removed, and the cells were washed twice with phosphate-buffered saline (PBS). The cells were then divided into four groups: (A) control group: only serum-free culture medium was added; (B) Yoda1(MCE, dissolved in DMSO) 10 μM group: 10 μM Yoda1 was added; (C) Yoda1 20 μM group: 20 μM Yoda1 was added; (D)Yoda1 30 μM group: 30 μM Yoda1 was added; After incubation for 24 h, cell viability was determined using the Cell Counting Kit-8(CCK-8) (Beyotime, CHN) solution, which was added to each well and incubated for 2 h. The absorbance was measured at 450 nm using a microplate reader (Thermo Multiskan MK3; Thermo Fisher Scientific).

### Annexin V-FITC/PI double staining

The cells were seeded into a six-well plate for 12 h and then treated with Yoda1 in the absence or presence of BAPTA or NAC for 24 h. Cells were washed twice with cold PBS and then resuspended in 500μL binding buffer. After adding Annexin V (5μL) and PI (5μL), the cells were incubated in the dark for 15 min. Then data were analyzed by the FACS Calibur flow cytometer (BD Biosciences).

### Western blot

Total proteins were extracted from the cells with RIPA lysis buffer on ice for 30 min. The protein concentration was determined by the bicinchoninic acid (BCA) assay at 562 nm, and an equal amount of protein samples was separated by 12.5% sodium dodecyl sulfate–polyacrylamide gel electrophoresis (SDS-PAGE) and then transferred to a polyvinylidene fluoride (PVDF) membrane. The membrane was incubated with the indicated antibodies against Bcl-2, Bax, cleaved-caspase3/9, β-actin, VDAC Antibody, and Cytochrome c Antibody overnight at 4 °C, followed by respective secondary antibodies at 37 °C for 1 h. Finally, the bands were visualized by enzyme-linked chemiluminescence according to the manufacturer’s protocol (ECL; Bio-Rad). Original cropped western blot gel image was shown in Additional file 2.

### Measurement of the mitochondrial membrane potential (MMP)

The MMP of the cells was assessed using the JC-10 Assay kit (Beyotime, CHN) in line with the manufacturer's protocol. Briefly, after the indicated treatments, cells were collected and incubated with JC-10 solution (1 mL) at 37 °C for 20 min and centrifuged at 600 × g for 3 min at 4˚C. Gently washed in PBS and resuspended in 1X incubation buffer (provided in the assay kit) twice. Finally, the MMP was determined by flow cytometry. The JC-10 polymer/monomer fluorescence ratio was used to quantify the changes in MMP.

### ROS detection

The cells were implanted in twenty-four-well plates and treated with Dichlorodihydrofluorescein diacetate (DCFH-DA) (Beyotime, CHN) diluted with the serum-free medium under 1:1000 to a final concentration of 10 μmol/L. The plate was incubated for 30 min at 37 °C in a cell incubator. The cells were washed with a serum-free cell culture medium three times to remove the DCFH-DA that did not enter the cells fully, and the images were captured by fluorescence microscopy. Image J software was used to analyze the fluorescence intensity.

### *Detection of cytosolic Ca*^*2*+^*concentration*

The cells were exposed to 30 μM Yoda1 for 24 h and took an appropriate amount of Fluo-4 AM (Beyotime, CHN) mother solution and diluted to the 0.5-5 μM working solution with PBS. Removed the cell culture solution, washed the cells twice, and added the Fluo-4 AM. Incubate the 37 °C cells in the incubator for 30 min in the dark. Then wash with PBS three times. After washing, you can consider another 30 min to ensure Fluo-4 AM is wholly converted into Fluo-4 in the cell. The fluorescence intensity was measured by flow cytometer with excitation source and 488 and 525 nm emission, respectively.

### Mitochondrial protein extraction

First, collect the cells (please ensure that the number of cells must be large so that enough mitochondria can be extracted), wash the cells twice with PBS, blow down the cells with PBS, collect them into a centrifuge tube, and centrifuge at 900 g for 5 min. Discard the supernatant, add 1 mL of Lysis Buffer at 4 °C to resuspend the cells, transfer the cell suspension to a glass homogenizer, and grind it thoroughly; then pour the homogenate into an EP tube, centrifuge at 1000 g at 4 °C 5 min. Take the supernatant, transfer it to a new EP tube, centrifuge at 1000 g for 5 min at 4 °C. Remove the supernatant, transfer it to a new EP tube, centrifuge at 1200 g for 10 min at 4 °C. The clear part can be used as cytoplasmic protein. The residue is mitochondria, then add 0.5 mL Wash Buffer to the mitochondrial pellet to resuspend the mitochondrial pellet, centrifuge at 1000 g for 5 min at 4 °C. Take the supernatant into a new EP tube, Centrifuge at 1200 g at 4 °C for 10 min. Discard the supernatant, add 50μL of Store Buffer to the pellet mix well, and store at -80 °C.

#### Statistic analysis

Data are presented as the means ± standard deviation of six independent experiments. Data were analyzed using GraphPad Prism 5.0 software (GraphPad Software, Inc., La Jolla, CA, USA). Statistical significance was evaluated by one-way analysis of variance (ANOVA). The result of *P* < 0.05 was considered as statistically significant difference and *P* < 0.01 as greatly significant.

## Results

### The expression of Piezo1 in VSMCs

**Fig. 1:**
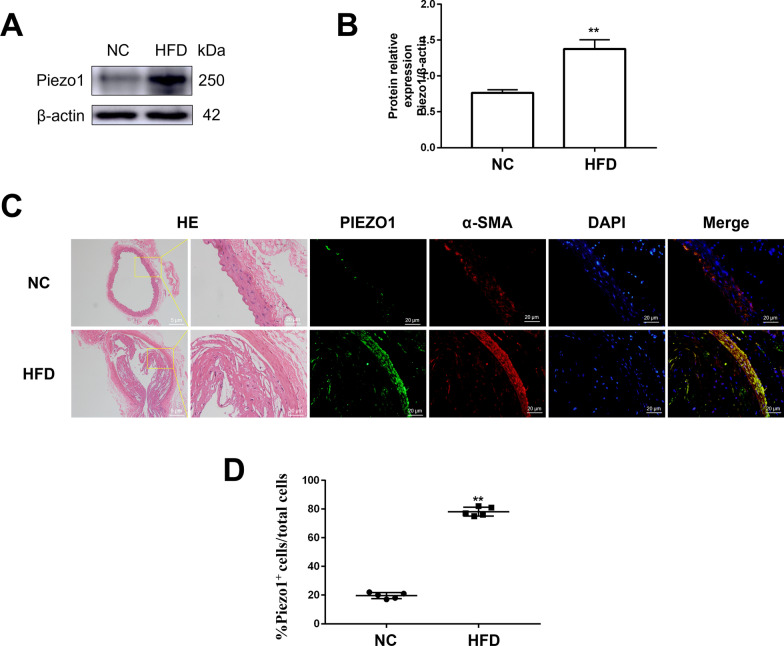
The expression of Piezo1 in VSMCs. (**A**)Detecting the expression level of Piezo1 in mouse vascular tissues by Western blot analysis. (**B**) Relative expression statistics of Western blot analysis. (**C, D**) The expression and localization of vascular smooth muscle cells by immunofluorescence staining. *P < 0.05 and **P < 0.01 vsersus Control

In vivo, western blot was used to detect the expression of Piezo1 in the aorta of each group, and the localization and expression of Piezo1 in each group were evaluated by immunofluorescence. The results showed that compared with the NC group, the expression of Piezo1 in the HFD group was significantly increased in atherosclerotic plaques (*P* < 0.05). Simultaneously, double staining showed that most Piezo1 positive cells were α-SMA positive, proving that Piezo1 was mainly expressed in VSMC in plaques (Fig. 1).

### *Piezo1 induced VSMCs apoptosis by activating the mitochondrial apoptotic pathway*.

**Fig. 2:**
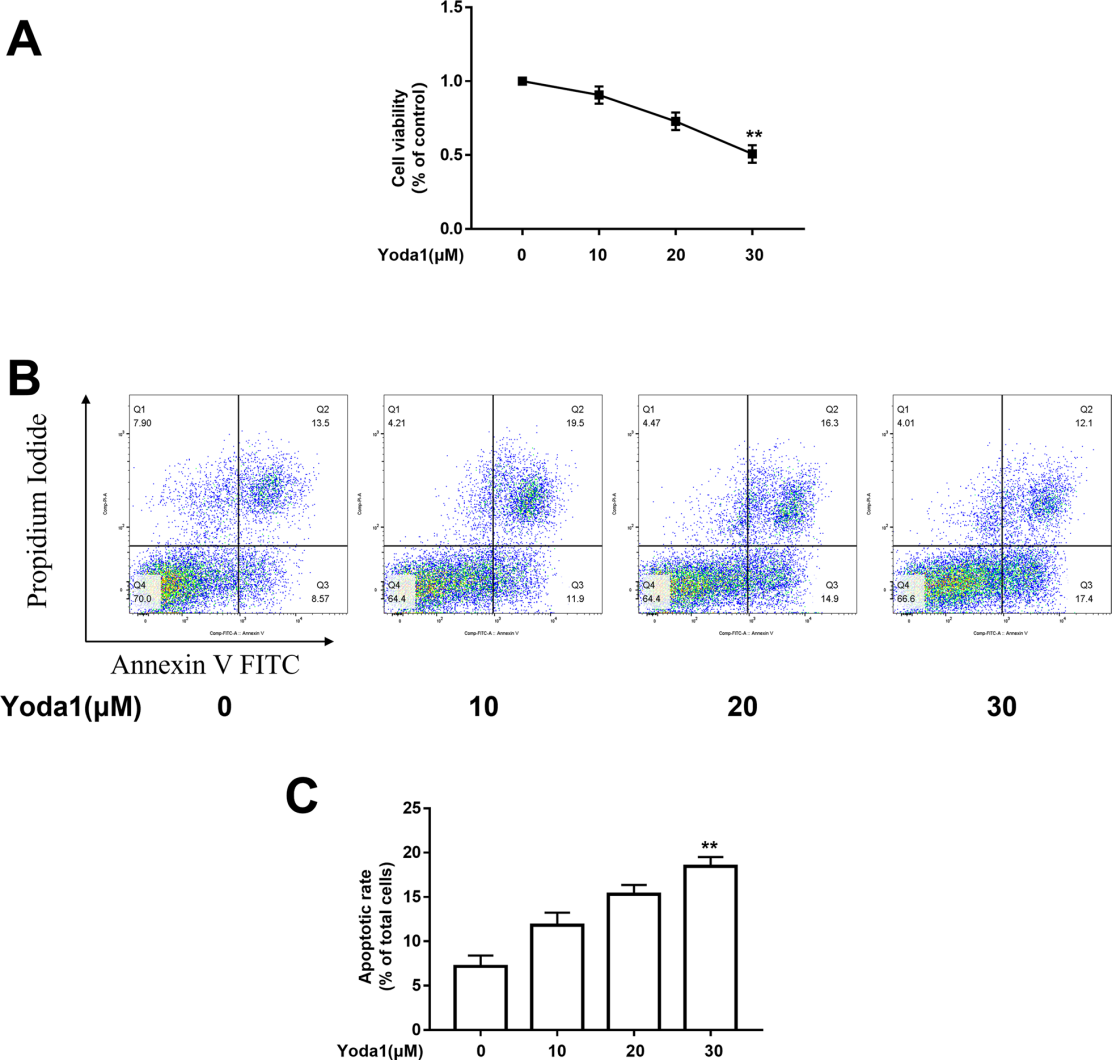

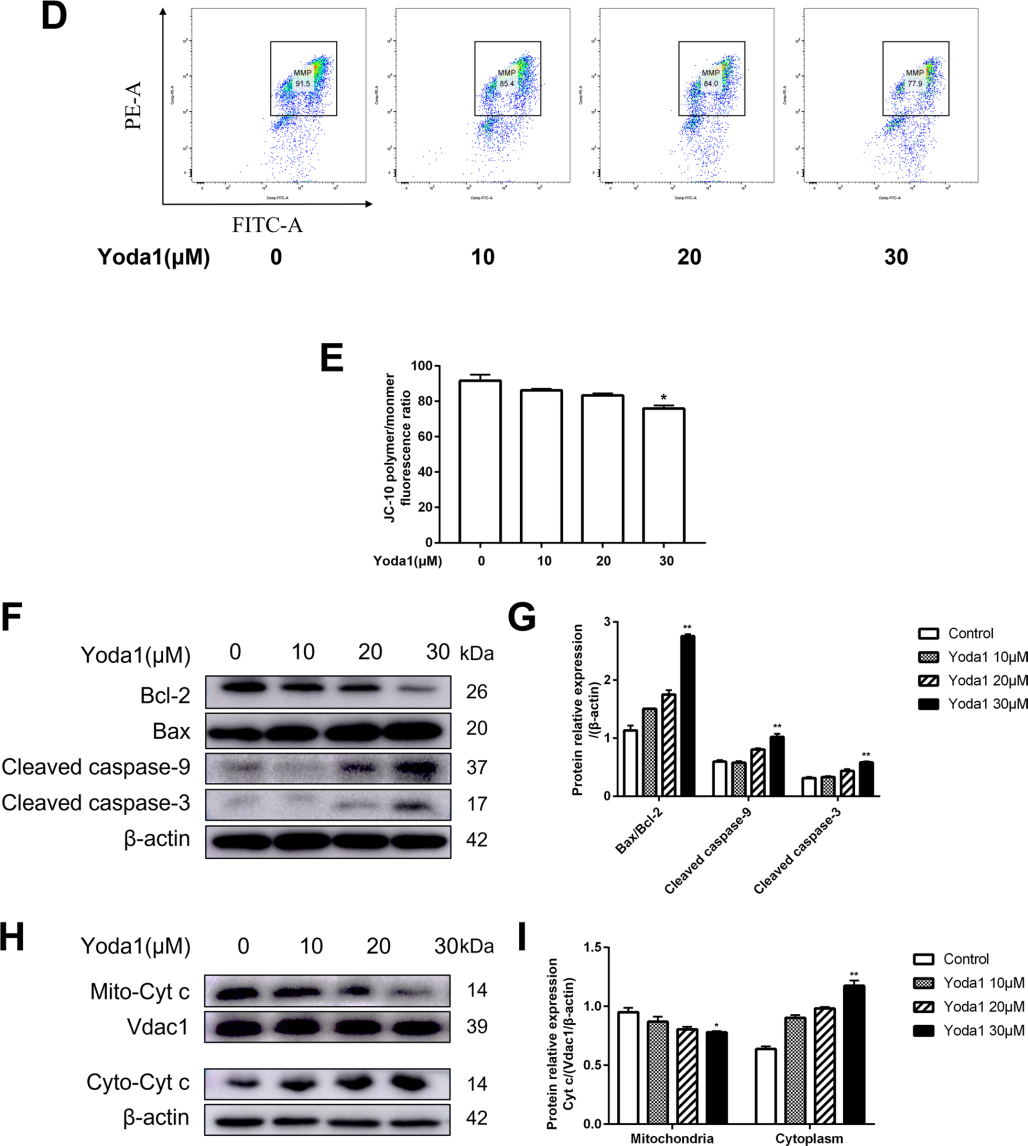
Piezo1 induced VSMCs apoptosis by activating the mitochondrial apoptotic pathway. Cells were treated with Yoda1 (10, 20, 30 μm) for 24 h. (**A**) The detection of cell viability. (**B, C**) The apoptosis rate of each group. (**D**) MMP of each group was determined. (**E**) Quantitative analysis of the MMP. (**F, H**) Western blot analysis of several apoptotic-related protein expression levels. (**G, I**) Relative expression statistics of Western blot analysis. *P < 0.05 and **P < 0.01 versus Control

CCK-8 was used to detect changes in cell viability of each group under the stimulation of Yoda1 (Piezo1 agonist). The apoptosis rate of VSMCs of each group was determined by AnnexinV-FITC/PI flow cytometry. Western blot was used to detect the expression of apoptosis-related proteins in each group. The results showed that compared with the control group, the cell viability of the Yoda1 group was significantly decreased (*P* < 0.05), and the cell apoptosis rate was significantly increased (*P* < 0.05). Cell morphological changes were shown in Additional file 1: Supplementary Figure S2. The protein expression of mitochondrial apoptosis-related factors Bax, cleaved caspase-9, cleaved caspase-3 was increased, Bcl-2 was decreased (*P* < 0.05); mitochondrial membrane potential was also reduced with the increase of Yoda1 concentration (*P* < 0.05), The expression of Cyto-Cyt c was increased while Mito-Cyt c showed an opposite trend (*P* < 0.05)(Fig. 2).

### *Piezo1 increases cytosolic Ca*^*2*+^*and ROS levels in VSMCs*

**Fig. 3:**
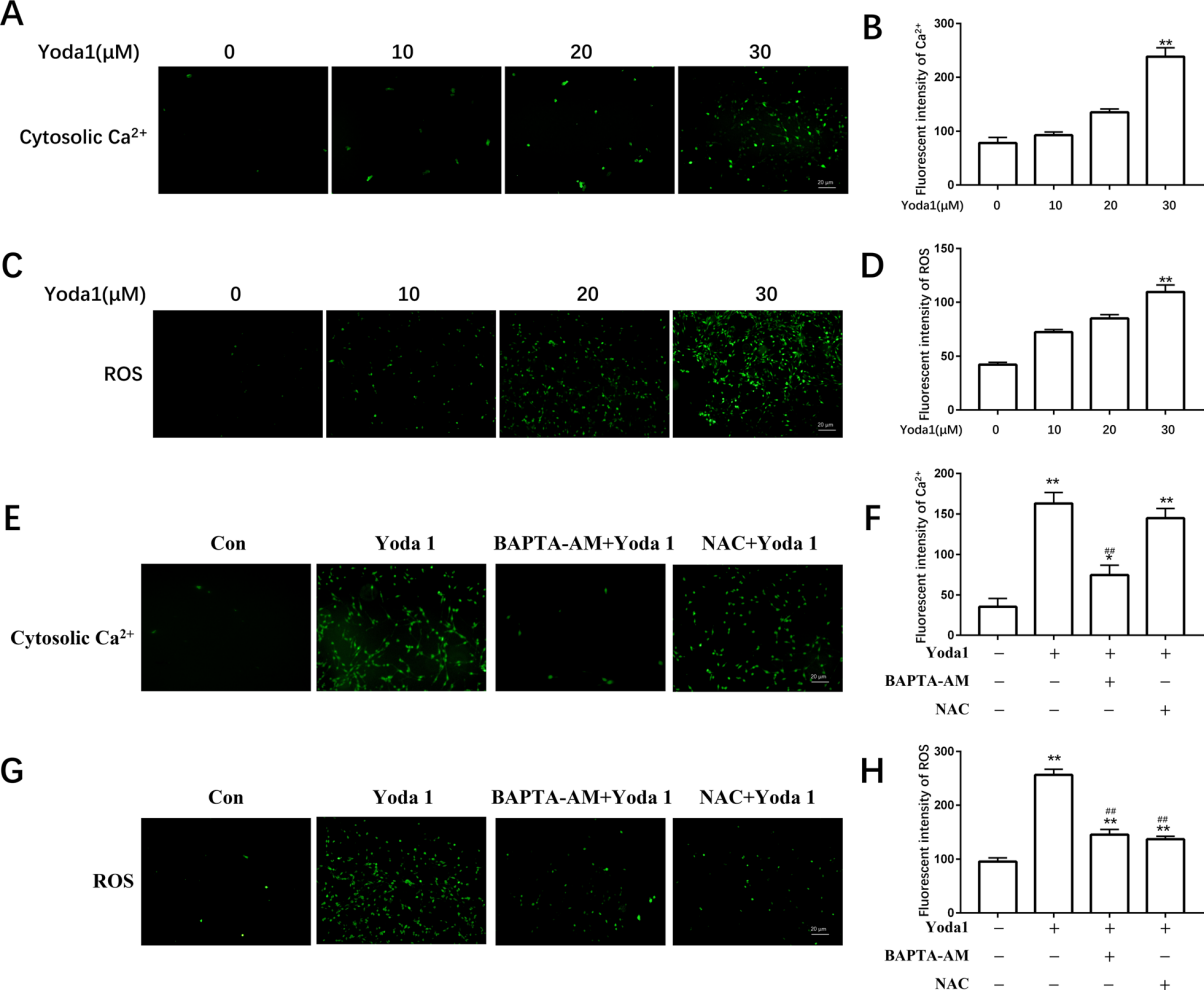
Effect of Piezo1 on intracellular ROS and cytosolic Ca^2+^ levels. (**A, E**) Detecting intracellular Ca^2+^ levels. (**B, F**) Quantitative analysis of intracellular Ca^2+^ levels. (**C, G**) The result of ROS levels was determined. (**D**, **H**) Quantitative analysis of ROS.BAPTA-AM (10 μM), NAC (40 mM). *P < 0.05 and **P < 0.01 versus Control. ^#^*P* < 0.05 and ^##^*P* < 0.01 vs. cell treated with Yoda1(30 μM) alone

After stimulated VSMCs with Yoda1, calcium ion probe and ROS probe were used to detect intracellular calcium ion concentration and ROS level changes. The results showed that the calcium ion concentration and ROS level in the Yoda1 group were increased(*P* < 0.05). While pretreatment of VSMCs with BAPTA-AM and NAC in vitro, BAPTA-AM could inhibit the increase in intracellular Ca^2+^ concentration, and ROS level was caused by Yoda1, but NAC only inhibited the level of ROS and had no significant effect on the intracellular calcium ion concentration (*P* < 0.05)(Fig. 3).

### *Piezo1 induces mitochondrial-dependent VSMCs apoptosis *via* Ca*^*2*+^*overload and ROS accumulation.*

**Fig. 4:**
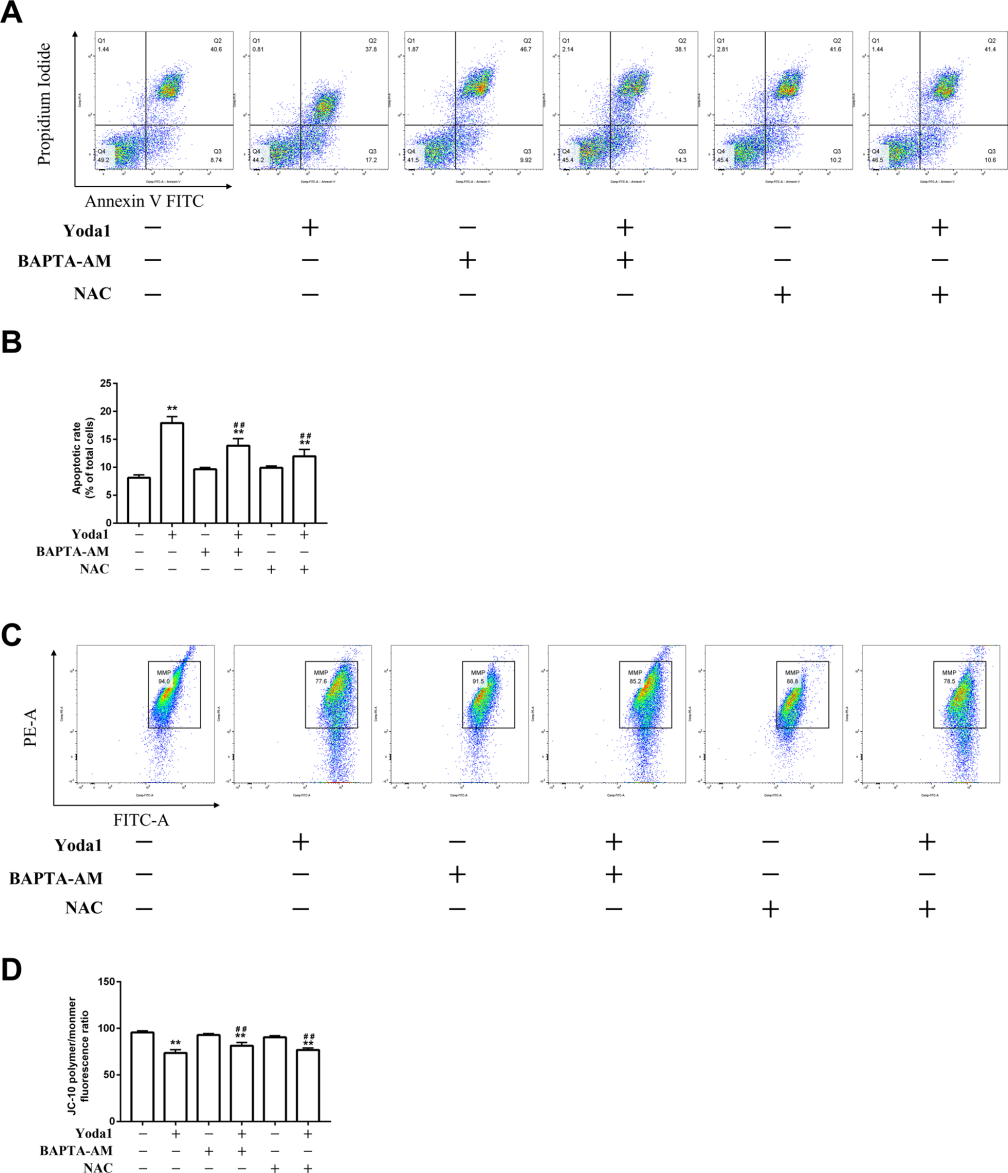

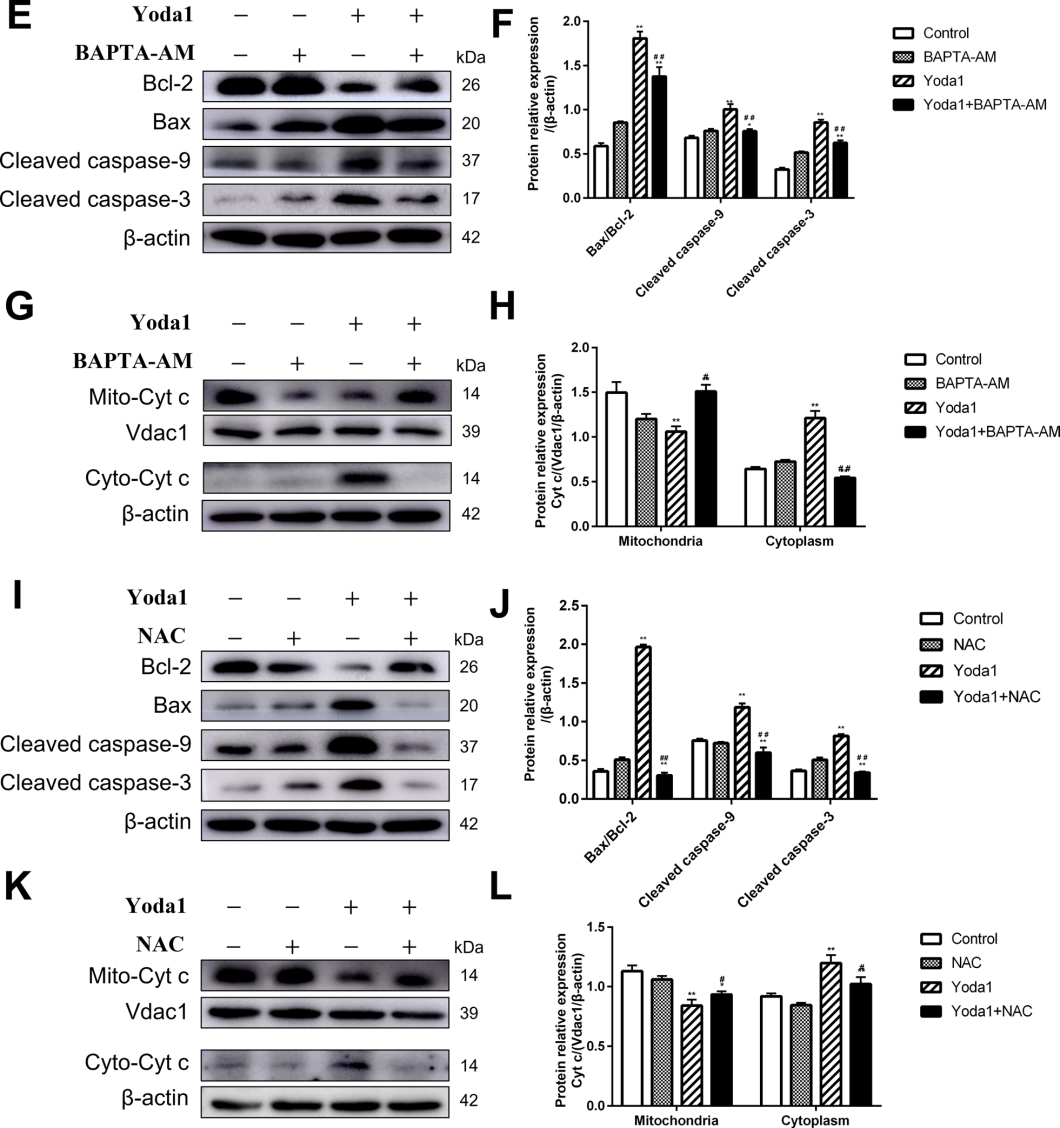
Piezo1 induces mitochondrial-dependent VSMCs apoptosis via Ca^2+^ overload and ROS accumulation. (**A, B**) The fraction of apoptotic cells was assessed. (**C, D**) The MMP of VSMCs was assessed. (**E–H**) Western blot analysis of apoptotic related proteins expression level. (**I–L**) Western blot analysis of related proteins expression level. BAPTA-AM (10 μM), NAC (40 mM). *P < 0.05 and **P < 0.01 vsersus Control. ^#^*P* < 0.05 and ^##^*P* < 0.01 vs. cell treated with Yoda1(30 μM) alone

With BAPTA-AM and NAC pretreatment, the apoptosis rate of VSMCs was lower than that in the Yoda1 group (*P* < 0.05), and it weakened the expression of proapoptotic molecules Bax, cleaved caspase-9, and cleaved caspase-3 in the Yoda1 group, and increased the expression of anti-apoptotic molecule Bcl-2 (*P* < 0.05). Meanwhile, the mitochondrial membrane potential was restored (*P* < 0.05), the transport of Cytochrome c from mitochondria into the cytoplasm was reduced (*P* < 0.05) (Fig. 4).

## Discussion

Piezos is mainly composed of two members, Piezo1 and Piezo2. Piezo1 is widely distributed in erythrocytes, lung epithelial cells, fibroblasts, chondrocytes, etc., while the expression of Piezo2 is mainly confined to the nervous system. In addition, Piezo1 is also present on vascular endothelial cells and vascular smooth muscle cells, and relevant evidence suggests that Piezo1 has an important role in the cardiovascular system. Piezo1 is a non-selective mechanosensitive ion channel activated by shear stress in local blood flow and tension in the cell membrane [10]. It plays a vital role in vascular development, blood pressure regulation, and blood vessel remodeling and participates in the regulation of cell apoptosis. Piezo1 on vascular endothelial cells senses shear forces as early as the embryonic stage and helps ensure the correct alignment and morphology of endothelial cells. In addition, Piezo1 can modulate the significant reduction in vessel wall diameter and thickness in chronic hypertensive mice. Opening of Piezo1 has been found to increase intracellular Ca^2+^ levels, which may lead to the activation of calcium-dependent transglutaminase in smooth muscle cells, ultimately leading to vascular remodeling[11–13]. Studies revealed that Piezo1 promotes atherosclerosis formation by regulating the NF-κB pathway in vascular endothelial cells and can activate membrane-type matrix metalloproteinase(MMP)-1 and MMP-2 to promote angiogenesis, thereby promoting unstable plaque formation[14, 15]. However, it has not been determined whether Piezo1 induced VSMCs apoptosis. In the present study, we found that Piezo1 induced mitochondrial-dependent VSMCs apoptosis via Ca^2+^ overload and ROS accumulation.

Numerous studies have confirmed that vulnerable plaque leads to the sudden occurrence of cardiovascular events, and fibrous cap rupture is the leading cause of vulnerable plaque rupture and cardiovascular events.[16–18]. Moreover, the apoptosis of vascular smooth muscle cells in advanced atherosclerosis is crucial in rupturing fibrous caps and thrombosis. Therefore, the treatment of vascular smooth muscle cell apoptosis is essential for the clinical prevention and treatment of atherosclerosis and cardiovascular events. This study found increased Piezo1 expression in the vascular wall of aortic blood vessels from ApoE^−/−^ mice. Besides, we identify the expression of Piezo1 in VSMCs of plaques was also increased compared to normal tissues. This phenomenon reminds us that Piezo1 may play an essential role in regulating VSMCs apoptosis in atherosclerotic plaques.

In vitro, Piezo1 activation was recreated under static conditions using Yoda1, the only known agonist of Piezo1. Some studies have shown that Yoda1 was the downstream signal event of Piezo1 mediated by calcium inserted into cells and red blood cells[19, 20]. Hence, we used Yoda1 as a tool for activating the Piezo1. We observed the cell viability decreased in Yoda1 treated in a dose-dependent manner by CCK-8 assay. Meanwhile, we detected the apoptosis rate by the Annexin V-FITC/PI double staining. We found that exposure of VSMCs to 30 μM Yoda1 for 24 h significantly promoted apoptosis. Therefore, we selected this concentration and time to carry on the subsequent experiment to explore the underlying mechanism.

It is generally accepted that apoptosis is a complex cell death process, mainly divided into mitochondrial pathways, death receptor pathways, and endoplasmic reticulum stress pathways [21]. Mitochondria are important bioenergy sources and biosynthetic centers in cells, mitochondrial calcium overload leads to opening of mitochondrial permeability transition pore, generate and sequester toxic ROS in response to oxidative stress, which can result in mitochondrial dysfunction and apoptotic cell death. Recent studies suggested that calcium overload leads to mitochondria-mediated apoptosis, promoting VSMCs apoptosis and endothelial dysfunction, which can alter the homeostasis of the cardiovascular system, also causing plaque instability and acute coronary syndrome[22, 23]. Wang et al*.* [24] observed the activation of Piezo1 sensitizes cells to TRAIL-mediated apoptosis through mitochondrial outer membrane permeability. To further determine the potential mechanism of how Piezo1 induces VSMCs apoptosis, we focused on the mitochondrial apoptosis pathway. The present study results revealed that Yoda1 decreased the expression of Bax and increased the expression of Bcl-2, resulting in a decreased Bcl-2/Bax ratio in VSMCs. Besides, the loss of the MMP is accompanied by the release of Cyt c from the mitochondria to the cytosol, which eventually leads to the activation of apoptotic enzymes caspase-9 and caspase-3[25]. Next, we examined the change of MMP in VSMCs. The results showed that Yoda1 led to a decline in MMP and promoted the release of Cyt c from mitochondria into the cytosol. A reduction in the Bcl-2/Bax ratio can induce the loss of the MMP[26]. The present study supported the hypothesis that Piezo1 may induce VSMCs apoptosis by activating the mitochondrial apoptotic pathway.

The activation of Piezo1 can cause calcium influx[27, 28]. Clempus et al. [29] proposed that Ca^2+^ overload in the cytoplasm leads to depolarization of mitochondria, thereby promoting ROS accumulation. Slaven et al. demonstrated the Docosahexaenoic acid-induced apoptosis in vascular smooth muscle cells is triggered by Ca^2+^-dependent induction of oxidative stress[30]. In the present study, we found after treatment with Yoda1, the levels of ROS and cytoplasmic Ca^2+^ in vascular smooth muscle cells increased significantly. Therefore, we hypothesized that Ca^2+^ might enter the cell through Piezo1 activated and consequently damage the mitochondria, which induces the production of a significant quantity of ROS and apoptosis. BAPTA-AM, a well-established intracellular calcium ion chelator, is often used in research related to Ca^2+^[31, 32]. The mitochondria and NADPH oxidase produce ROS[33]. N-Acetylcysteine (NAC) is an antioxidant that can reduce intracellular ROS concentrations by scavenging free radicals[34, 35]. As expected, NAC and BAPTA attenuated the up-regulation of Bax, down-regulation of Bcl-2, the release of Cyt c, loss of MMP, activation of caspase-9, and caspase-3 caused by Yoda1 in VSMCs. Meanwhile, BAPTA-AM significantly inhibited the increase of Ca^2+^ and ROS. In contrast, NAC has little effect on the cytoplasmic Ca^2+^, which indicated that Yoda1 induced cytoplasmic Ca^2+^ overload increased ROS level and ultimately induced VSMCs apoptosis.

Meanwhile, other types of VSMCs death in atherosclerosis have been identified, such as autophagy, ferroptosis, and pyroptosis. However, the potential mechanism of VSMCs death induced by Piezo1 has not been investigated yet. Thus, more efforts are needed to address this issue.

## Conclusion

**Fig. 5:**
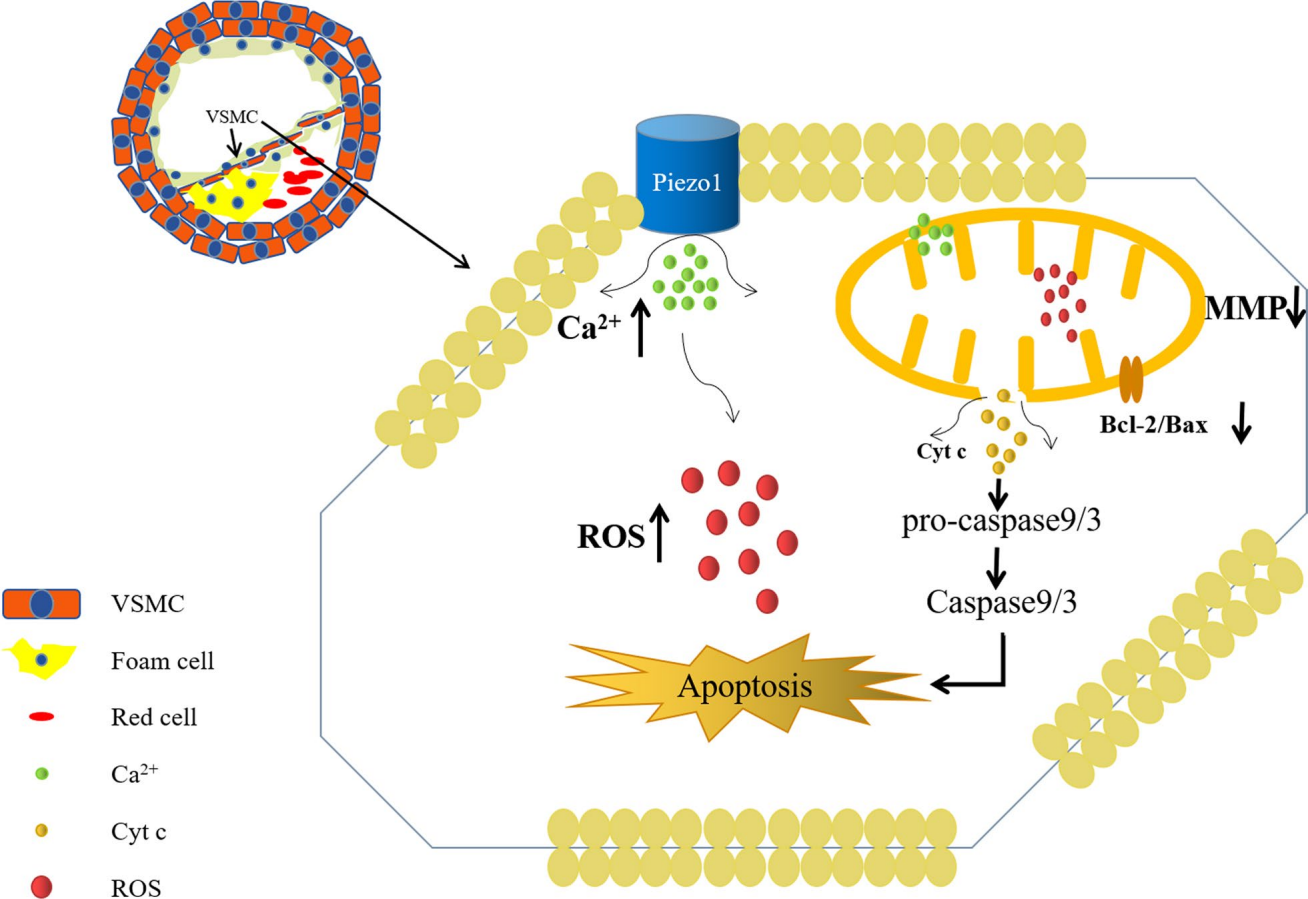
Piezo1 promotes ROS production, calcium overload, loss of mitochondrial membrane potential, Cyt c release, up-regulation of Bax/Bcl-2 ratio, activation of caspase-3 and caspase-9, and ultimately induces mitochondrial-dependent VSMCs apoptosis

Finally, it may be concluded the activation of Piezo1 contributed to the overload of intracellular Ca^2+^ and mitochondrial damage, which elevated the ROS level and eventually led to the initiation of cell apoptosis (Fig. 5). The present study may promote further investigation on the application of Piezo1 inhibition, and it may become a potential target for the prevention or therapy of vascular diseases.

## Supplementary Information

**Additional file 1**. Supplementary figures and table.

**Additional file 2**. Original cropped western blot gel image.

## Data Availability

The datasets used and/or analysed during the current study available from the corresponding author on reasonable request.

## References

[1] Clarke MC, Figg N, Maguire JJ, Davenport AP, Goddard M, Littlewood TD, Bennett MR (2006). Apoptosis of vascular smooth muscle cells induces features of plaque vulnerability in atherosclerosis. Nat Med.

[2] Bennett MR, Sinha S, Owens GK (2016). Vascular smooth muscle cells in atherosclerosis. Circ Res.

[3] Grootaert M, Moulis M, Roth L, Martinet W, Vindis C, Bennett MR, De Meyer G (2018). Vascular smooth muscle cell death, autophagy and senescence in atherosclerosis. Cardiovasc Res.

[4] Mercer J, Figg N, Stoneman V, Braganza D, Bennett MR (2005). Endogenous p53 protects vascular smooth muscle cells from apoptosis and reduces atherosclerosis in ApoE knockout mice. Circ Res.

[5] Rössig L, Dimmeler S, Zeiher AM (2001). Apoptosis in the vascular wall and atherosclerosis. Basic Res Cardiol.

[6] Bai X, Bouffard J, Lord A, Brugman K, Sternberg PW, Cram EJ, Golden A: Caenorhabditis elegans PIEZO channel coordinates multiple reproductive tissues to govern ovulation. *Elife* 2020, 9. 10.7554/eLife.53603 PMC7340503 32490809

[7] Ilkan Z, Wright JR, Goodall AH, Gibbins JM, Jones CI, Mahaut-Smith MP (2017). Evidence for shear-mediated Ca2+ entry through mechanosensitive cation channels in human platelets and a megakaryocytic cell line. J Biol Chem.

[8] Liang GP, Xu J, Cao LL, Zeng YH, Chen BX, Yang J, Zhang ZW, Kang Y (2019). Piezo1 induced apoptosis of type II pneumocytes during ARDS. Respir Res.

[9] Sun Y, Leng P, Song M, Li D, Guo P, Xu X, Gao H, Li Z, Li C, Zhang H (2020). Piezo1 activates the NLRP3 inflammasome in nucleus pulposus cell-mediated by Ca2+/NF-κB pathway. Int Immunopharmacol.

[10] Coste B, Mathur J, Schmidt M, Earley TJ, Ranade S, Petrus MJ, Dubin AE, Patapoutian A (2010). Piezo1 and Piezo2 are essential components of distinct mechanically activated cation channels. Science.

[11] Murthy SE, Dubin AE, Patapoutian A (2017). Piezos thrive under pressure: mechanically activated ion channels in health and disease. Nat Rev Mol Cell Biol.

[12] Retailleau K, Duprat F, Arhatte M, Ranade SS, Peyronnet R, Martins JR, Jodar M, Moro C, Offermanns S, Feng Y (2015). Piezo1 in smooth muscle cells is involved in hypertension-dependent arterial remodeling. Cell Rep.

[13] Wang YY, Zhang H, Ma T, Lu Y, Xie HY, Wang W, Ma YH, Li GH, Li YW (2019). Piezo1 mediates neuron oxygen-glucose deprivation/reoxygenation injury via Ca2+/calpain signaling. Biochem Biophys Res Commun.

[14] Albarrán-Juárez J, Iring A, Wang S, Joseph S, Grimm M, Strilic B, Wettschureck N, Althoff TF, Offermanns S (2018). Piezo1 and G(q)/G(11) promote endothelial inflammation depending on flow pattern and integrin activation. J Exp Med.

[15] Kang H, Hong Z, Zhong M, Klomp J, Bayless KJ, Mehta D, Karginov AV, Hu G, Malik AB (2019). Piezo1 mediates angiogenesis through activation of MT1-MMP signaling. Am J Physiol Cell Physiol.

[16] Badimon L, Vilahur G (2014). Thrombosis formation on atherosclerotic lesions and plaque rupture. J Intern Med.

[17] Ahmadi A, Leipsic J, Blankstein R, Taylor C, Hecht H, Stone GW, Narula J (2015). Do plaques rapidly progress prior to myocardial infarction? The interplay between plaque vulnerability and progression. Circ Res.

[18] Pelisek J, Eckstein HH, Zernecke A (2012). Pathophysiological mechanisms of carotid plaque vulnerability: impact on ischemic stroke. Arch Immunol Ther Exp (Warsz).

[19] Lacroix JJ, Botello-Smith WM, Luo Y (2018). Probing the gating mechanism of the mechanosensitive channel Piezo1 with the small molecule Yoda1. Nat Commun.

[20] Wang S, Chennupati R, Kaur H, Iring A, Wettschureck N, Offermanns S (2016). Endothelial cation channel PIEZO1 controls blood pressure by mediating flow-induced ATP release. J Clin Invest.

[21] Bock FJ, Tait S (2020). Mitochondria as multifaceted regulators of cell death. Nat Rev Mol Cell Biol.

[22] Li P, Wang J, Zhao X, Ru J, Tian T, An Y, Tang L, Bai Y (2020). PTEN inhibition attenuates endothelial cell apoptosis in coronary heart disease via modulating the AMPK-CREB-Mfn2-mitophagy signaling pathway. J Cell Physiol.

[23] Yu S, Zhang L, Liu C, Yang J, Zhang J, Huang L (2019). PACS2 is required for ox-LDL-induced endothelial cell apoptosis by regulating mitochondria-associated ER membrane formation and mitochondrial Ca(2+) elevation. Exp Cell Res.

[24] Hope JM, Lopez-Cavestany M, Wang W, Reinhart-King CA, King MR (2019). Activation of piezo1 sensitizes cells to TRAIL-mediated apoptosis through mitochondrial outer membrane permeability. Cell Death Dis.

[25] Kang S, Kim K, Noh JY, Jung Y, Bae ON, Lim KM, Chung JH (2016). Simvastatin induces the apoptosis of normal vascular smooth muscle through the disruption of actin integrity via the impairment of RhoA/Rac-1 activity. Thromb Haemost.

[26] Giménez-Cassina A, Danial NN (2015). Regulation of mitochondrial nutrient and energy metabolism by BCL-2 family proteins. Trends Endocrinol Metab.

[27] Cahalan SM, Lukacs V, Ranade SS, Chien S, Bandell M, Patapoutian A: Piezo1 links mechanical forces to red blood cell volume. *Elife* 2015, 4. 10.7554/eLife.07370 PMC4456639 26001274

[28] Li J, Hou B, Tumova S, Muraki K, Bruns A, Ludlow MJ, Sedo A, Hyman AJ, McKeown L, Young RS (2014). Piezo1 integration of vascular architecture with physiological force. Nature.

[29] Clempus RE, Griendling KK (2006). Reactive oxygen species signaling in vascular smooth muscle cells. Cardiovasc Res.

[30] Crnkovic S, Riederer M, Lechleitner M, Hallström S, Malli R, Graier WF, Lindenmann J, Popper H, Olschewski H, Olschewski A (2012). Docosahexaenoic acid-induced unfolded protein response, cell cycle arrest, and apoptosis in vascular smooth muscle cells are triggered by Ca^2+^-dependent induction of oxidative stress. Free Radic Biol Med.

[31] Kip SN, Hunter LW, Ren Q, Harris PC, Somlo S, Torres VE, Sieck GC, Qian Q (2005). [Ca2+]i reduction increases cellular proliferation and apoptosis in vascular smooth muscle cells: relevance to the ADPKD phenotype. Circ Res.

[32] Fu Z, Fan Q, Zhou Y, Zhao Y, He Z (2019). Elimination of intracellular calcium overload by BAPTA-AM-loaded liposomes: a promising therapeutic agent for acute liver failure. ACS Appl Mater Interfaces.

[33] Maryanovich M, Gross A (2013). A ROS rheostat for cell fate regulation. Trends Cell Biol.

[34] Zafarullah M, Li WQ, Sylvester J, Ahmad M (2003). Molecular mechanisms of N-acetylcysteine actions. Cell Mol Life Sci.

[35] Jin L, Ni J, Tao Y, Weng X, Zhu Y, Yan J, Hu B (2019). N-acetylcysteine attenuates PM2.5-induced apoptosis by ROS-mediated Nrf2 pathway in human embryonic stem cells. Sci Total Environ.

