# Supplementary material for: Agonist-induced Piezo1 activation promote mitochondrial-dependent apoptosis in vascular smooth muscle cells
Source: BMC Cardiovasc Disord. 2022 Jun 24;22:287. doi: 10.1186/s12872-022-02726-2 (PMC9233385; doi:10.1186/s12872-022-02726-2)

Panel 1 represents western blot analysis shown in Fig 1A, Piezo1.
Panel 2 represents western blot analysis shown in Fig 1A, β-actin.
Panel 3 represents western blot analysis shown in Fig 2F, Bcl-2.
Panel 4 represents western blot analysis shown in Fig 2F, Bax.
Panel 5 represents western blot analysis shown in Fig 2F Caspase-9.
Panel 6 represents western blot analysis shown in Fig 2F, Caspase-3.
Panel 7 represents western blot analysis shown in Fig 2F, β-actin.
Panel 8 represents western blot analysis shown in Fig 2H, Mito-Cytc.
Panel 9 represents western blot analysis shown in Fig 2H, Vdac1.
Panel 10 represents western blot analysis shown in Fig 2H, Cyto-Cytc.
Panel 11 represents western blot analysis shown in Fig 2H, β-actin.
Panel 12 represents western blot analysis shown in Fig 4E, Bcl-2.
Panel 13 represents western blot analysis shown in Fig 4E, Bax.
Panel 14 represents western blot analysis shown in Fig 4E Caspase-9.
Panel 15 represents western blot analysis shown in Fig 4E, Caspase-3.
Panel 16 represents western blot analysis shown in Fig 4E, β-actin.
Panel 17 represents western blot analysis shown in Fig 4G, Mito-Cytc.
Panel 18 represents western blot analysis shown in Fig 4G, Vdac1.
Panel 19 represents western blot analysis shown in Fig 4G, Cyto-Cytc.
Panel 20 represents western blot analysis shown in Fig 4G, β-actin.
Panel 21 represents western blot analysis shown in Fig 4I, Bcl-2.
Panel 22 represents western blot analysis shown in Fig 4I, Bax.
Panel 23 represents western blot analysis shown in Fig 4I Caspase-9.
Panel 24 represents western blot analysis shown in Fig 4I, Caspase-3.
Panel 25 represents western blot analysis shown in Fig 4I, β-actin.
Panel 26 represents western blot analysis shown in Fig 4K, Mito-Cytc.
Panel 27 represents western blot analysis shown in Fig 4K, Vdac1.
Panel 28 represents western blot analysis shown in Fig 4K, Cyto-Cytc.
Panel 29 represents western blot analysis shown in Fig 4K, β-actin.

Figure 1A Piezo1, β-actin

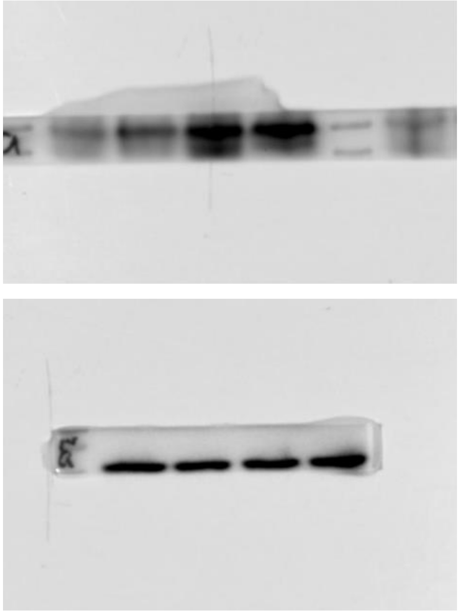

Figure 2F Bcl-2, Bax, Caspase-9, Caspase-3, β-actin

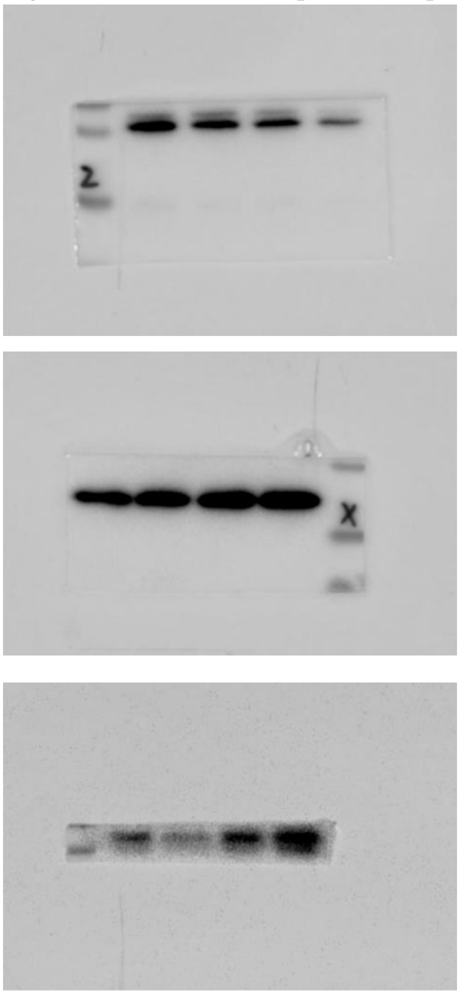

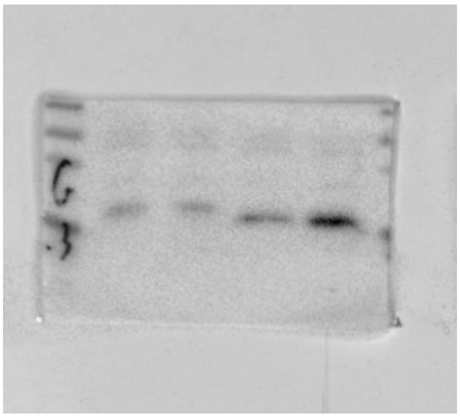

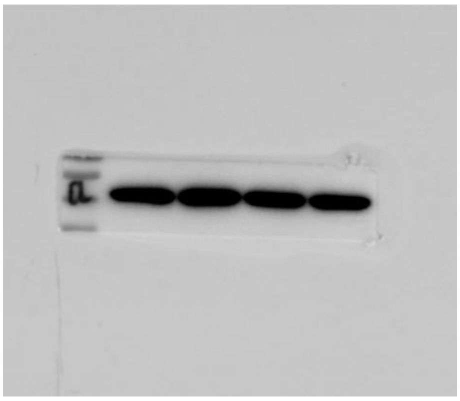

Figure 2H Mito-Cytc, Vdac1, Cyto-Cytc, β-actin

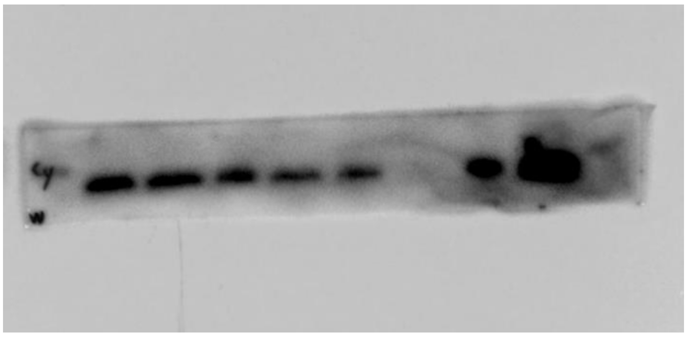

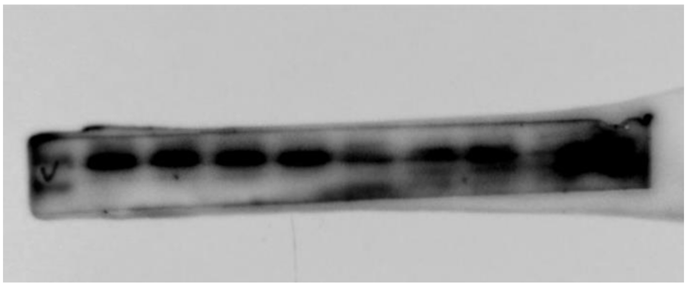

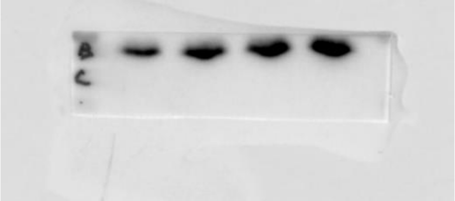

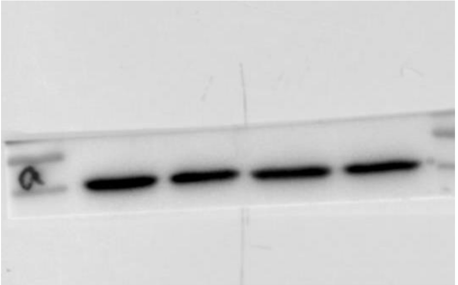

Figure 4E Bcl-2, Bax, Caspase-9, Caspase-3, β-actin

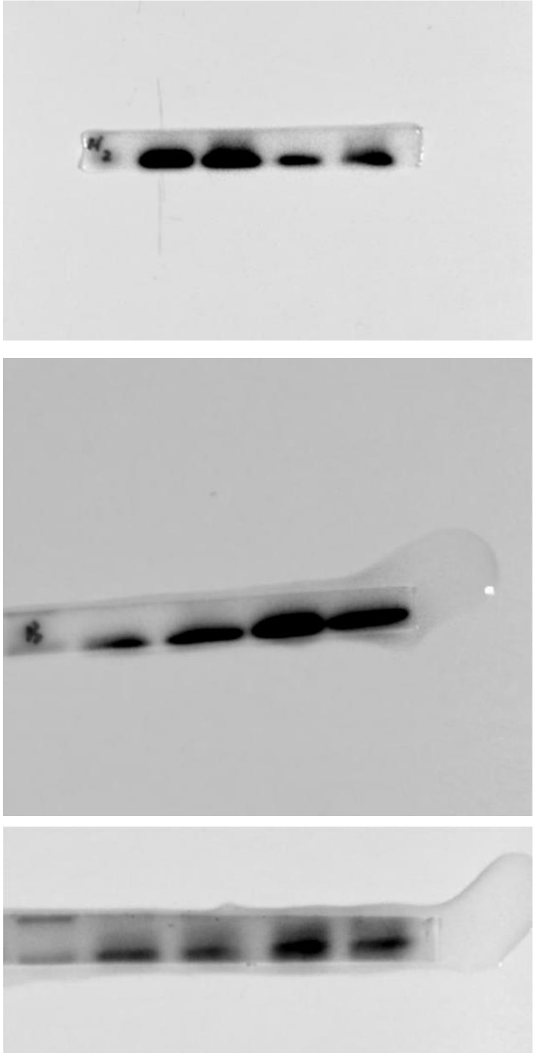

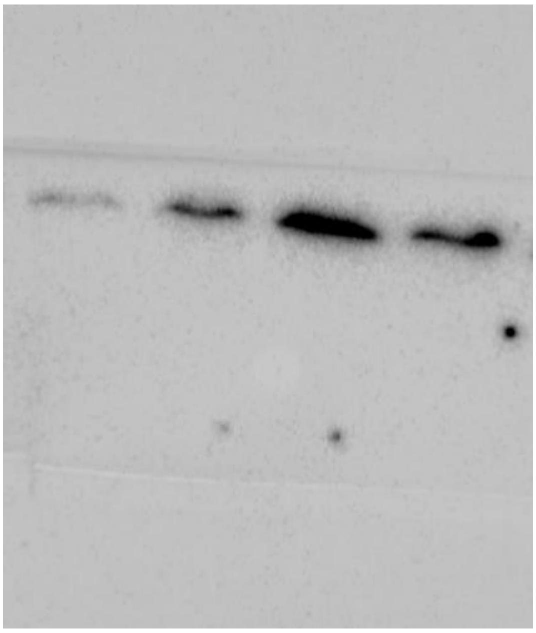

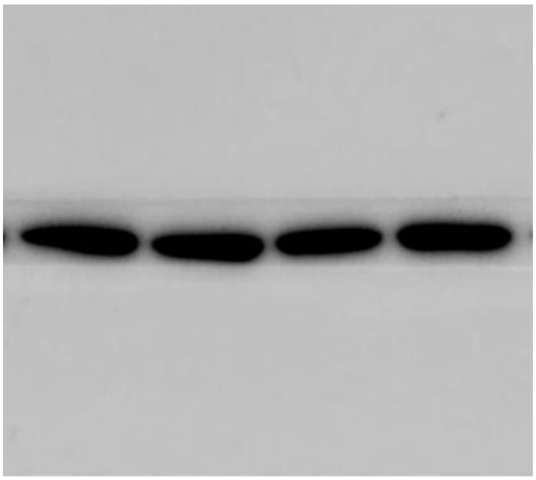

Figure 4G Mito-Cytc, Vdac1, Cyto-Cytc, β-actin

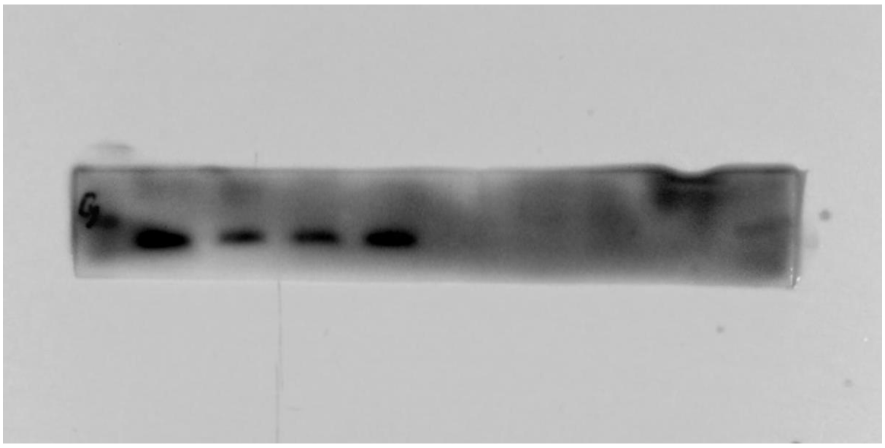

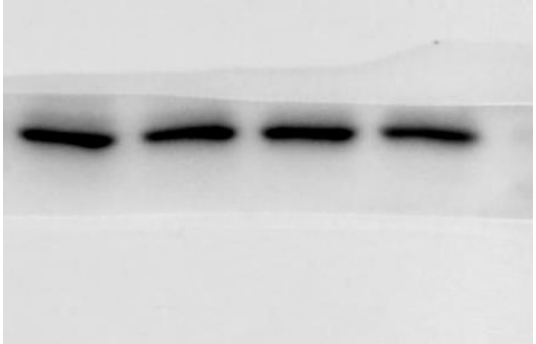

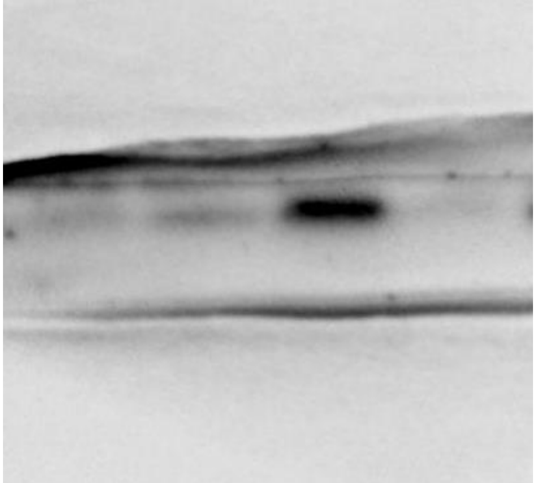

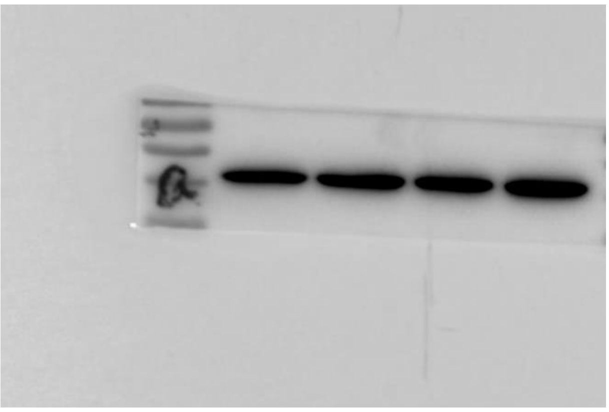

Figure 4I Bcl-2, Bax, Caspase-9, Caspase-3, β-actin

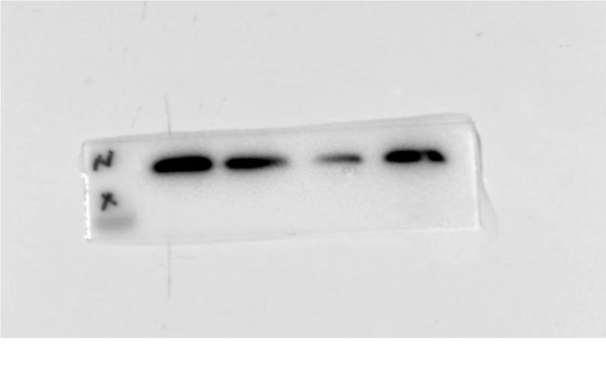

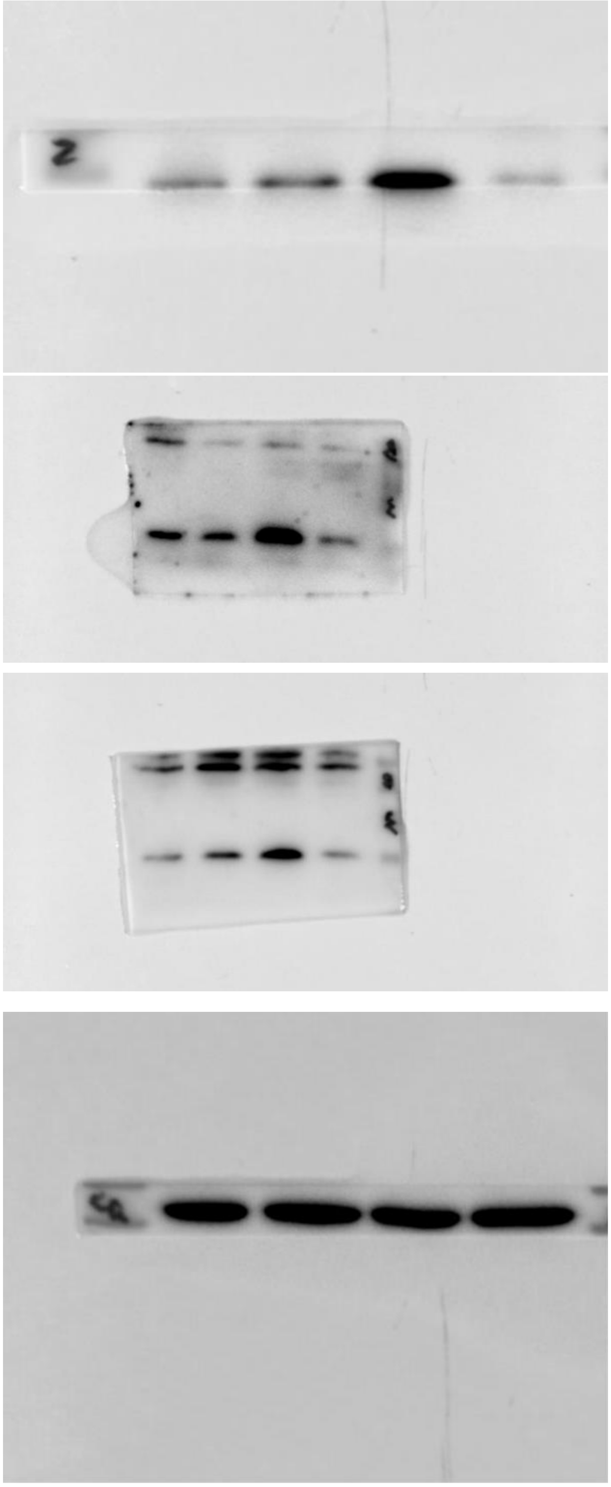

Figure 4K Mito-Cytc, Vdac1, Cyto-Cytc, β-actin

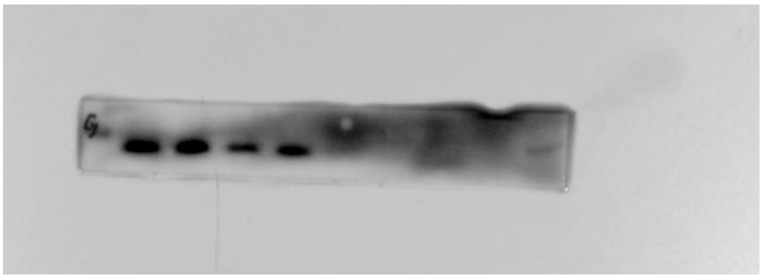

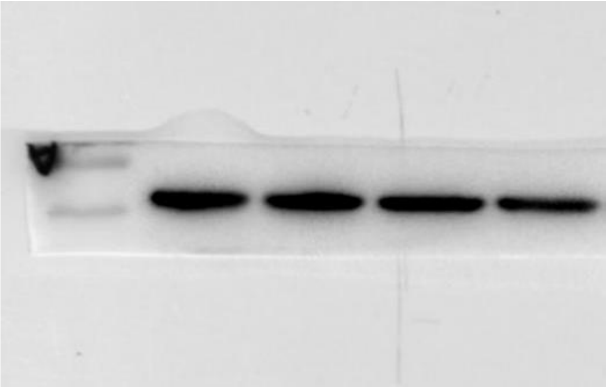

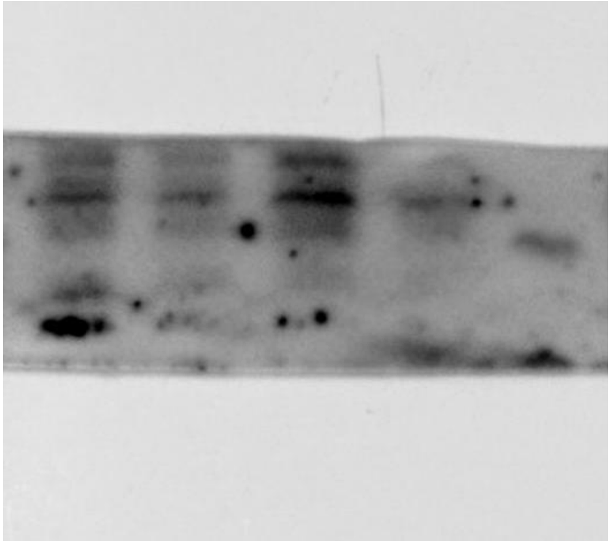

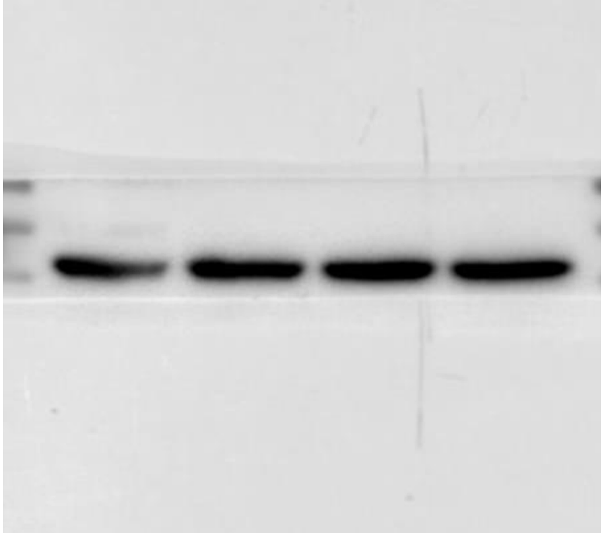

Supplement: Supplementary file 1 — Additional file 1. Supplementary figures and table. [file 12872_2022_2726_MOESM1_ESM.pdf]
