# Supplementary material for: Agonist-induced Piezo1 activation promote mitochondrial-dependent apoptosis in vascular smooth muscle cells
Source: BMC Cardiovasc Disord. 2022 Jun 24;22:287. doi: 10.1186/s12872-022-02726-2 (PMC9233385; doi:10.1186/s12872-022-02726-2)
Supplement: Supplementary file 2 — Additional file 2. Original cropped western blot gel image. [file 12872_2022_2726_MOESM2_ESM.docx]

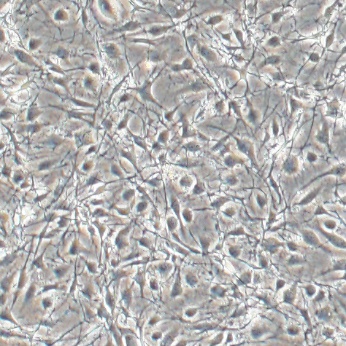

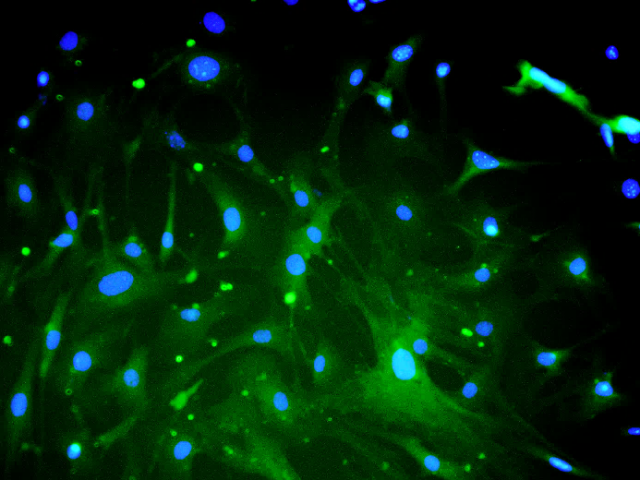


**Supplementary fig. S1**α-SMA Fluorescence Staining of Smooth Muscle Cells. The VSMCs were obtained from the thoracic aorta of male, eight-week-old C57BL/6J mice. Briefly, the thoracic aortas were harvested from the C57 mice, add collagenase to the petri dish with the blood vessels and put them into a CO_2_ incubator to digest for about 5-8 minutes. Place the Petri dish under a stereo microscope, peel off the adventitia of the blood vessel with sterile forceps and chop it into 1mm^2^ tissue pieces. After putting in collagenase, put it into the incubator to digest for 2-3 hours. The digestion was stopped with 1 mL of complete medium, and then transferred to a centrifuge tube, centrifuged at 900 rpm for 8 minutes, the supernatant was discarded, and resuspended in 1 mL of medium containing 30% FBS. After about 2 weeks, the tissue block was removed, the cells were passaged, and 3-5 passages of VSMCs were taken for subsequent experiments.


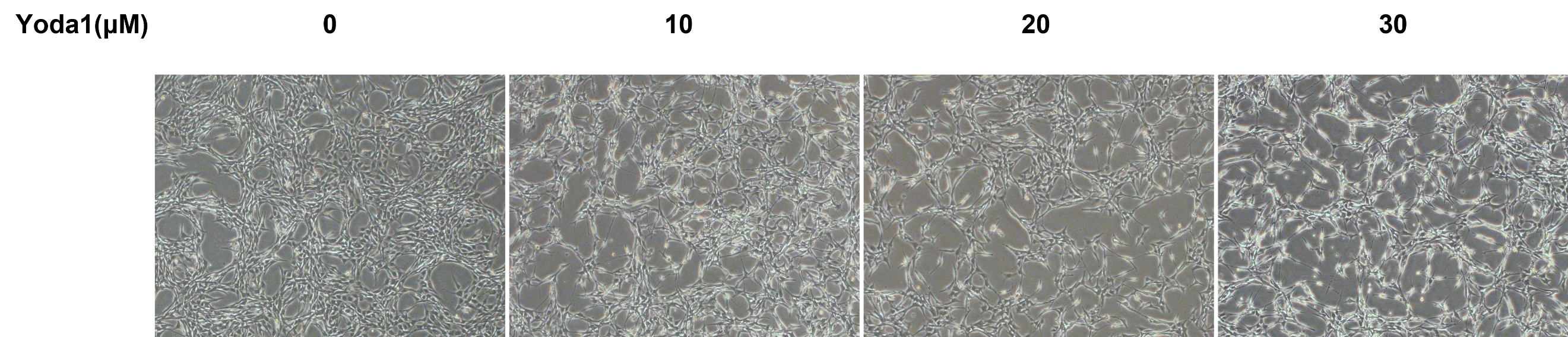


**Supplementary fig. S2** Cell morphological changes. As the concentration of Yoda1 increases, the intercellular space becomes larger, the VSMCs become elongated into a spindle shape, and the apoptotic cells become brighter and float in the supernatant.


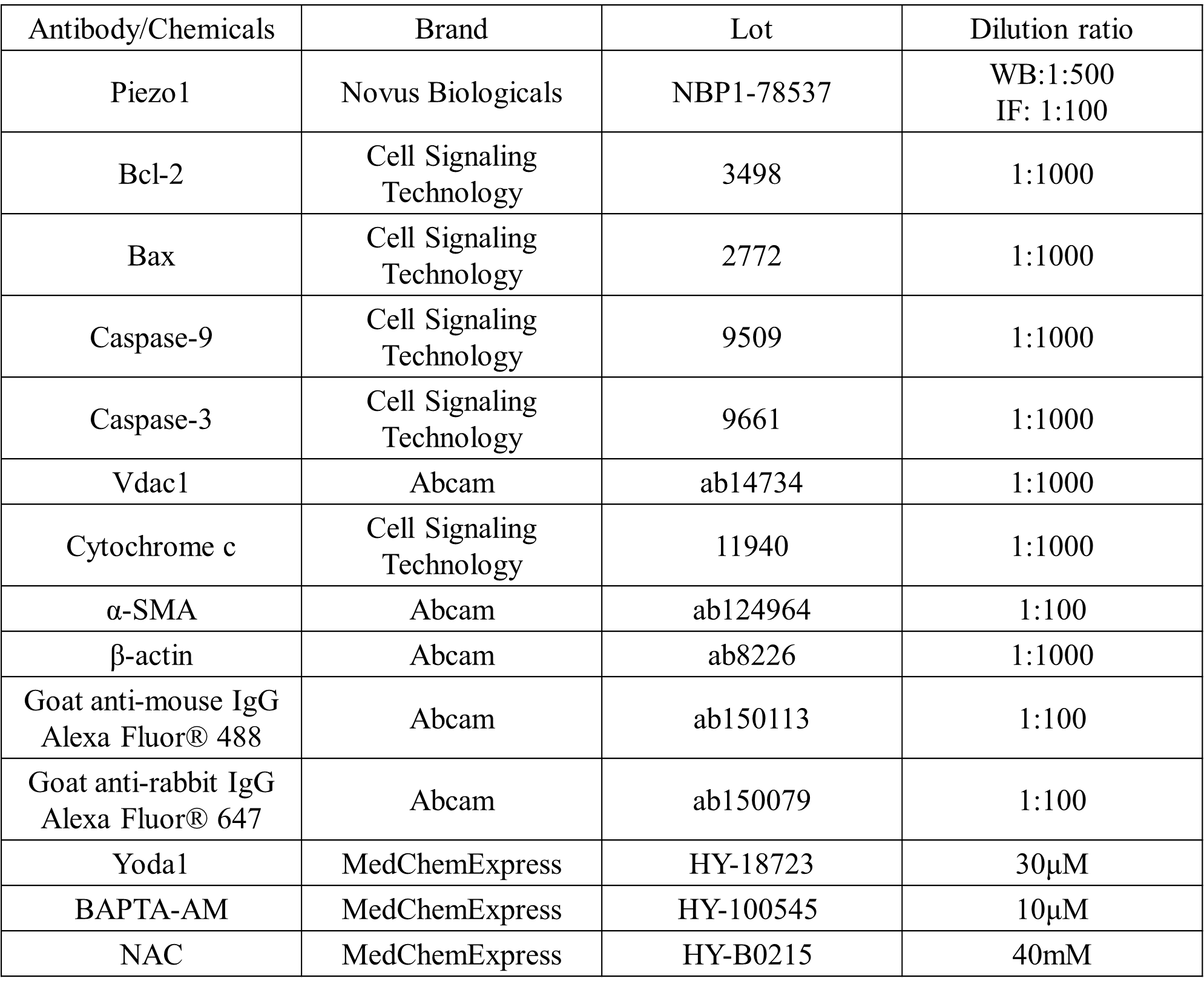


**Supplementary table. S1** Detailed information on antibodies and chemicals
